# Comprehensive genome sequence analysis of the devastating tobacco bacterial phytopathogen *Ralstonia solanacearum* strain FJ1003

**DOI:** 10.3389/fgene.2022.966092

**Published:** 2022-08-22

**Authors:** Kun Chen, Yuhui Zhuang, Lihui Wang, Huaqi Li, Taijie Lei, Mengke Li, Meijia Gao, Jiaxian Wei, Hao Dang, Ali Raza, Qiang Yang, Yasir Sharif, Huan Yang, Chong Zhang, Huasong Zou, Weijian Zhuang

**Affiliations:** ^1^ State Key Laboratory of Ecological Pest Control for Fujian and Taiwan Crops, Fujian Agriculture and Forestry University, Fuzhou, China; ^2^ College of Plant Protection, Fujian Agriculture and Forestry University, Fuzhou, China; ^3^ Oil Crops Research Institute, College of Agriculture/Center of Legume Crop Genetics and Systems Biology, Fujian Agriculture and Forestry University, Fuzhou, China; ^4^ College of Life Science, Fujian Agriculture and Forestry University, Fuzhou, China

**Keywords:** *Ralstonia solanacearum*, tobacco, bacterial wilt, disease resistance, effector proteins, genome sequencing, Rs_T3E_Hyp14

## Abstract

Due to its high genetic diversity and broad host range, *Ralstonia solanacearum*, the causative phytopathogen of the bacterial wilt (BW) disease, is considered a “species complex”. The *R. solanacearum* strain FJ1003 belonged to phylotype I, and was isolated from the Fuzhou City in Fujian Province of China. The pathogen show host specificity and infects tobacco, especially in the tropical and subtropical regions. To elucidate the pathogenic mechanisms of FJ1003 infecting tobacco, a complete genome sequencing of FJ1003 using single-molecule real-time (SMRT) sequencing technology was performed. The full genome size of FJ1003 was 5.90 Mb (GC%, 67%), containing the chromosome (3.7 Mb), megaplasmid (2.0 Mb), and small plasmid (0.2 Mb). A total of 5133 coding genes (3446 and 1687 genes for chromosome and megaplasmid, respectively) were predicted. A comparative genomic analysis with other strains having the same and different hosts showed that the FJ1003 strain had 90 specific genes, possibly related to the host range of *R. solanacearum*. Horizontal gene transfer (HGT) was widespread in the genome. A type Ⅲ effector protein (Rs_T3E_Hyp14) was present on both the prophage and genetic island (GI), suggesting that this gene might have been acquired from other bacteria *via* HGT. The Rs_T3E_Hyp14 was proved to be a virulence factor in the pathogenic process of *R. solanacearum* through gene knockout strategy*,* which affects the pathogenicity and colonization ability of *R. solanacearum* in the host. Therefore, this study will improve our understanding of the virulence of *R. solanacearum* and provide a theoretical basis for tobacco disease resistance breeding.

## Introduction

Bacterial wilt disease (BWD) caused by *Ralstonia solanacearum* is a devastating soil-borne disease (Cheng et al., 2021). Owing to its large genetic diversity, it is also known as the *R. solanacearum* species complex (RSSC). RSSC damages numerous economically important crops, such as tobacco (Xiao et al., 2018), peanut (Chen et al., 2021), tomato (Im et al., 2020), potato (Tan et al., 2019), and pepper (Xianyang et al., 2018), thus resulting in substantial economic losses. Upon infection, RSSC can invade the host plant’s vascular tissues of roots through the intercellular spaces, where it multiplies and secretes exopolysaccharides that block the vascular tissue, thus causing the death of the host plant (Xiaoqiang, 2018). RSSC also lurks in some asymptomatic hosts to prepare for its subsequent transmission (Genin and Denny, 2012). According to classical taxonomic classification, RSSC can be classified into four phylotypes (Ι, II, III, and IV) (Paudel et al., 2020). However, based on genomics and proteomics, RSSC was reclassified into three phylotypes (I, II, and III) (Prior et al., 2016). Particularly, phylotypes I and III belong to the first type, while phylotypes II and IV belong to the second and third types, respectively. This classification has been accepted by several scholars and used for further experimentations.

Tobacco (*Nicotiana tabacum L*.) is an economically important global crop and a model plant for transgenic research. BWD is the third most damaging factor affecting tobacco productivity after black root rot and black shank disease (Liu et al., 2007). *Ralstonia solanacearum* invades tobacco through root wounds or natural cracks and then continues to infect xylem tissue, resulting in blockage of the host vascular bundle system and finally wilting and death (ZHOU et al., 2012). Tobacco bacterial wilt is widely occurring in the main tobacco-growing areas of China. Its incidence rate is about 80%, and in extreme cases, the crop is out of production, resulting in great economic losses to farmers (Wang, 2018).

The virulence factors of *R. solanacearum* responsible for promoting BWD mainly include exopolysaccharides, plant cell wall degrading enzymes, type III secretion system (T3SS), and type IV secretion system (T4SS) (Genin & Denny, 2012). Among them, the type III effector proteins (T3Es) secreted by T3SS are vital in the pathogenesis of *R. solanacearum*. Among bacteria, *R. solanacearum* has the most T3Es; with 60–75 of them existing in a single *R. solanacearum* genome (Genin and Denny, 2012; Peeters et al., 2013). Due to the large number of T3Es, *R. solanacearum* has a larger host range than other bacteria (Coll and Valls, 2013). Nonetheless, most T3Es are virulent, with only a few avirulent ones (Landry et al., 2020).

The whole-genome study of *R. solanacearum* gave novel insights into this complex species for further investigations. Previously, since the whole genome of the strain GMI1000 was sequenced first (Salanoubat et al., 2002), it is also known as the model strain. To date, 291 strains have complete genome assemblies and annotations in the NCBI database (https://www.ncbi.nlm.nih.gov/genome/browse/#!/prokaryotes/490/). Most of them consist of only one chromosome, with only a few having an additional small plasmid. Tobacco was the host of both Rs10 and CQPS-1 among the sequenced strains (https://www.ncbi.nlm.nih.gov/genome/browse/#!/prokaryotes/490/).

Therefore, this study reports the genome sequence of the FJ1003 strain of *R. solanacearum* isolated from tobacco in Fujian Province of China and analyzes its evolution, comparative genomics, and specific genes. The functions of Rs_T3E_Hyp14 were also investigated. Our findings provide a theoretical basis for the control and pathogenic mechanism of tobacco BWD and enrich the genomic information of *R. solanacearum*.

## Materials and methods

### Strain preparation and extraction of genomic DNA

The highly pathogenic *R. solanacearum* strain “FJ1003” (Zhang et al., 2017; Zhang et al., 2019) was provided by Professor Liu Bo from the Fujian Academy of Agricultural Sciences, Fuzhou, China (isolated from tobacco growing areas in Fujian Province). The strain was cultured on the bacteria peptone glucose (BG) solid medium (peptone 1.0%, yeast extract 0.1%, casamino acid 0.1%, glucose 1.0%, agar 1.4% and pH 7.4), and 50 ml BG liquid medium (peptone 1.0%, yeast extract 0.1%, casamino acid 0.1%, glucose 1.0%, pH 7.4) was used for single clone culturing at 28°C, 200 rpm overnight. The bacterial genome extraction kit (TIANGEN, Beijing, China) was used to extract the genomic DNA from the bacterial culture solution of *R. solanacearum* for subsequent sequencing experiments.

### Genome sequencing and assembly

The constructed bacterial genome library was sequenced using the single-molecule real-time (SMRT) sequencing technology by Biomarker Biosciences Co., Ltd. (Beijing, China) (Eid et al., 2009; Faino et al., 2015). Low-quality filtering was performed on the original reads obtained by sequencing; reads with a read length less than 100 bp and average read quality less than 0.7 were removed. The longest reads were selected as seed reads, and the remaining reads were compared to seed reads. Seed reads are converted into highly accurate pre-assembled reads for genome assembly. The complete genome was obtained by assembling the filtered subreads using the MHAP software with the default parameter (Chin et al., 2013; Berlin et al., 2015). The splicing and assembly are carried out in an overlapping manner, and the result is in the form of a scaffold sequence. Mainly through the overlap-layout-consensus method, the rectified high-quality pre-assembled reads sequences were assembled *de novo.* The quality of the assembly was verified using a probabilistic algorithm to determine the final gene sequence, and the ends of the assembled sequence were trimmed to circularize the genome.

### Genomic component analysis

We used the Repeat Masker (Tarailo Graovac and Chen, 2009) software to screen the repeated sequences in the bacterial genome. All coding genes and non-coding RNAs were predicted using the Prodigal software (Hyatt et al., 2010). GeneWise (Birney et al., 2004) was used to predict the presence of pseudogenes in the genome. The prophages and genomic islands were predicted using the PhiSpy (Akhter et al., 2012) and IslandPath-DIOMB (Bertelli and Brinkman, 2018) software, respectively. Furthermore, the PILLERCR software (Edgar, 2007) was used to predict the Clustered Regularly Interspaced Short Palindromic Repeats (CRISPR) in the genome.

### Functional annotation

The predicted gene sequences were BLASTed using functional annotation databases like COG (Tatusov et al., 2000), KEGG (Kanehisa, 2004), GO (Ashburner et al., 2000), Swiss-Prot (Consortium, 2018), NR (Deng et al., 2006), and PHI (http://www.phi-base.org/index.jsp). The functions of the genes were analyzed using the COG, KEGG metabolic pathways, GO, and PHI enrichment analysis.

### Gene family and unique gene analysis

OrthoMCL (Li et al., 2003) software was used to cluster the protein sequences of FJ1003, and the reference genome (GMI1000, CMR15, UW551, PSI07, CQPS_1, RS10) was used to discover the unique gene families of the strain. Based on sequence similarity, OrthoMCL can classify a set of proteins, such as genome-wide proteins, into ortholog groups. A total BLAST database was built using the protein sequences. All these sequences were run using BLASTP against the local database to get the results with an e-value < 1e^−5^. The percentage match length of the alignments was calculated using a 50% cut-off value. All ortholog, in-paralog, and co-ortholog pairs, and their normalized weight values, were entered into the MCL program for clustering.

### Analysis of evolutionary relationship among species

The protein sequences of the endoglucanase genes from FJ1003 and the reference genome were obtained using the annotation information. All the protein sequences used to build the RS_T3E_Hyp14 phylogenetic tree were downloaded from the T3Es (https://iant.toulouse.inra.fr/bacteria/annotation/site/prj/T3Ev3/) and NCBI (https://www.ncbi.nlm.nih.gov/) database. The evolutionary history was inferred using the maximum likelihood method based on the JTT (Jones-Taylor-Thornton) matrix-based model (Jones et al., 1992). The tree with the highest log-likelihood was shown. The percentage of tree in which the related taxa were clustered together was shown next to the branches. Initial tree(s) for the heuristic search were automatically obtained by applying the neighbor-joining (NJ) and BioNJ algorithms to a matrix of pairwise distances estimated using the JTT model and then selecting the topology with a superior log-likelihood value. The tree was drawn to scale, with the branch lengths measured in the number of substitutions per site. All positions containing gaps and missing data were eliminated. Finally, evolutionary analysis was performed in MEGA7 (Kumar et al., 2016).

### Collinearity analysis

Collinearity analysis was performed using the Multiple Collinearity Scan Toolkit (MCScanX) (Wang et al., 2012) to find the collinear genes between FJ1003 and the reference genome. Using the FJ1003 genomic protein sequences as a database and the reference genomic protein sequences as queries, the FJ1003 and other reference genomic protein sequences were compared by BLASTP. Finally, the file obtained after BLASTP was used as an input in MCScanX with the default parameters to visualize the results.

### Construction of mutant and complementary strains of *Ralstonia solanacearum*

The construction of mutant and complementary strains was carried out following the method of GUI et al. (2021), with slight modifications. Using the genomic DNA of *R. solanacearum* as a template, the upstream 440 bp and downstream 469 bp fragments of Rs_T3E_Hyp14 were amplified by specific primers (F1:5′-TCAACCGCAGCTACATCAGCTGCATG-3′,R1:5′-GTTACCGGACAGCGAAGAGCCAGCATTTGTGCAACTCCGTTACTGAGATCG-3′,F2:5′-CGATCTCAGTAACGGAGTTGCACAAATGCTGGCTCTTCGCTGTCCGGTAAC-3′,R2:5′-AAGAGCCGCTCTTTGCACTCCGATCA-3′). The upstream and downstream fragments were linked by overlapping PCR, digested by restriction enzymes XbaI and SphI (Takara, Shiga, Japan), and then ligated into the PK18 vector (PK18-U-D) by T4 DNA ligase enzyme (Takara, Shiga, Japan) and transformed into *R. Solanacearum* FJ1003, by homologous recombination. The Rs_T3E_Hyp14 in *R. solanacearum* was removed, and the Rs_T3E_Hyp14 mutant (ΔRs_T3E_Hyp14) was obtained that was confirmed by specific primers. For the complementary strains of Rs_T3E_Hyp14, the fragment U-Rs_T3E_Hyp14-D was amplified from the genomic DNA of *R. solanacearum* by the F1 and R2 primers. The fragment “U-Rs_T3E_Hyp14-D” was ligated into the PK18 vector (PK18-U-Rs_T3E_Hyp14-D) by T4 DNA ligase enzyme and transformed into the mutant strain. The complementary strain (CRs_T3E_Hyp 14ΔRs_T3E_Hyp14) was obtained by homologous recombination.

### Inoculation of *Ralstonia Solanacearum* and investigation of disease index

The *R. solanacearum* was cultured in BG medium until OD600 = 0.5 and then resuspended with sterile water. A susceptible tobacco variety, Honghua Dajinyuan (HD), was grown in plastic pots filled with nutrient soil to four-leaf stage. A sterilized scalpel was taken and inserted into the pot, causing mechanical damage to the roots. Then, 5 ml of bacterial solution was added to each pot and continued to culture in a greenhouse at 28°C. According to the classification and investigation methods of tobacco diseases and insect pests in China (GB/T23222—2008) (China, 2008), the disease index of infected tobacco plants was investigated at different time points. Three days after inoculation with *R. solanacearum*, 1 g of stem samples were ground in 1 ml sterile water, applied on BG medium after gradient dilution, and the number of colonies was calculated.

## Results

### Genome sequencing, assembly, and annotations

Using the SMRT sequencing technique, we sequenced the *R. solanacearum* FJ1003 strain isolated from tobacco at an average depth of 150X-250X, and obtained 1.38 Gb of high-quality sequencing data. We obtained three scaffolds, one bacterial chromosome, and two bacterial plasmids with a total length of 5.90 MB using the MHAP (Chin et al., 2013; Berlin et al., 2015) software (Table 1). Upon comparing the sequencing data to the assembled genome, the scaffolds were close to QV50 (Supplementary Figure S1), suggesting that we assembled a complete genome sequence.

**TABLE 1:** Statistical results of genomic characteristics of FJ1003 strain.

Type	Chromosome	Megaplasmid	Small plasmid	Total
Coding gene	3446	1564	123	5133
miRNA	3	1	0	4
rRNA	306	102	0	408
tRNA	32	3	0	35
Pseudogene	8	8	5	21
Size (bp)	3,753,705	1,996,333	146,771	5,896,809

**FIGURE 1:**
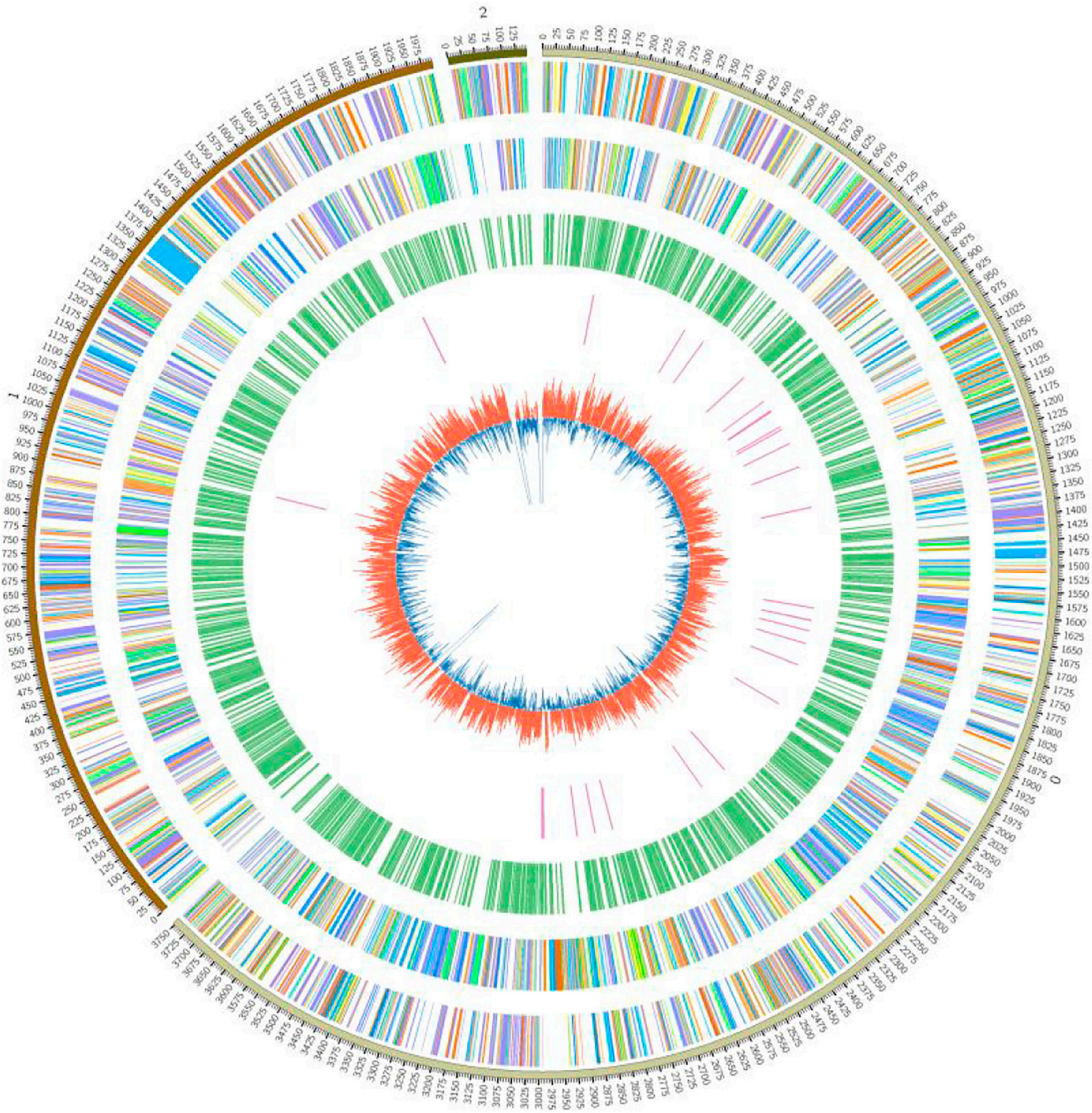
Circular map showing the genome of the *R. solanacearum* strain FJ1003. The outermost circle indicates the marker of genome size, with each scale of 0.1 Mb; the second and third circles indicate the genes on the positive and negative strands of the genome, respectively, and different colors represent the different functional categories of COG; the fourth circle represents repetitive sequences, and the fifth circle is tRNAs. The innermost layer indicates the GC content.

The (G + C) content in prokaryotes is generally higher than in eukaryotes. The (G + C) content of the FJ1003 sequenced genome was ∼67%. The statistical results of the genomic characteristics are presented in Table 1. In short, the FJ1003 genome contained 5133 coding genes, of which 3446, 1564, and 123 genes were located on the chromosome, the megaplasmid, and the small plasmid, respectively. The FJ1003 genome also had 447 non-coding RNAs (4 miRNAs, 35 tRNAs, and 408 rRNAs) and 21 pseudogenes. CRISPR is an important component of the immune system in prokaryotes that helps resist foreign plasmids and phage sequences. It holds the potential to recognize and silence the invading functional elements (Zhong et al., 2021). The predicted CRISPR sequences in the sequenced species are described in Supplementary Table S1. At the same time, based on the sequence and assembly results of the genome, we developed a circular genome map (Figure 1).

**FIGURE 2:**
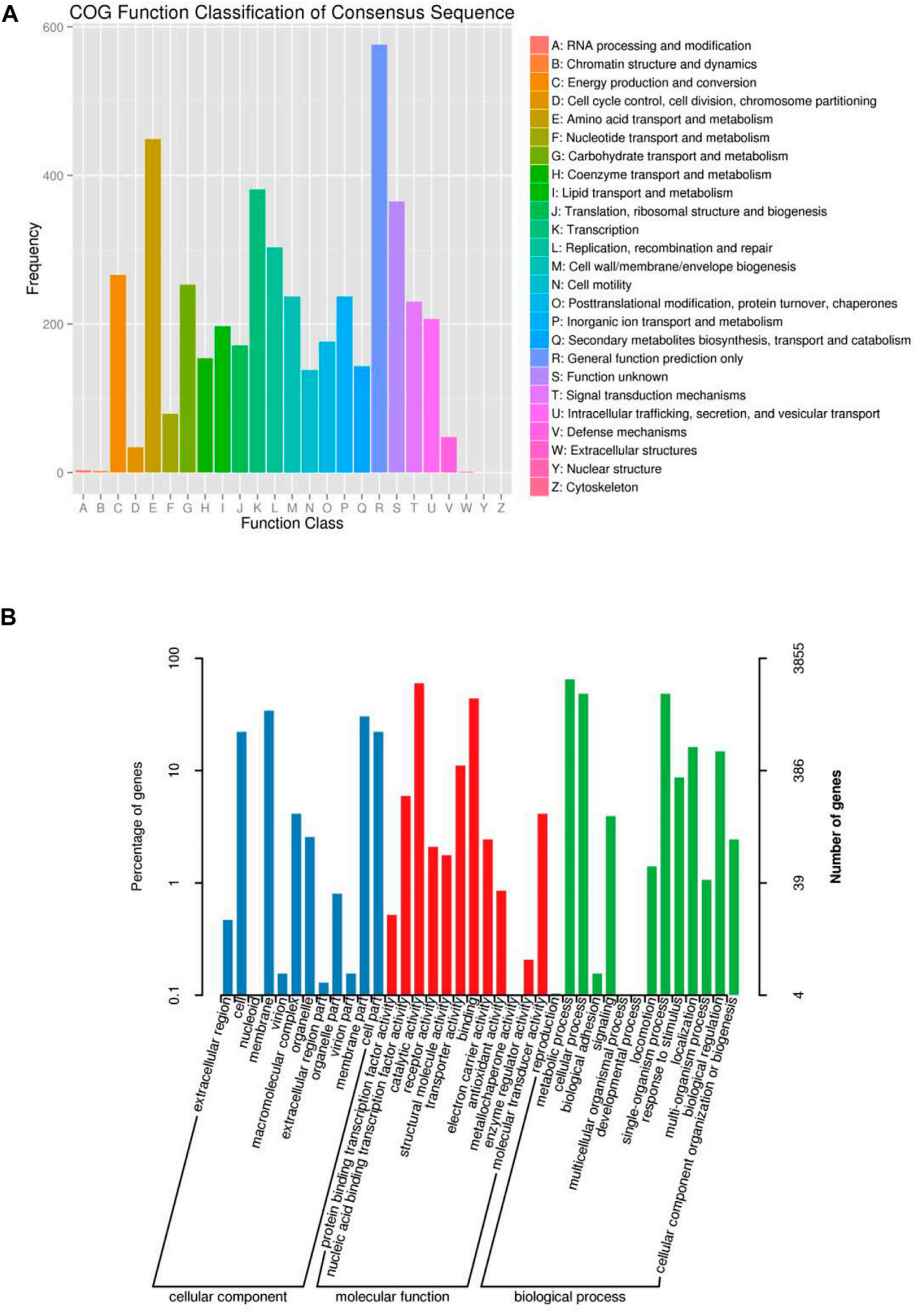
COG and GO functional categories of protein-coding genes in the *R. solanacearum* FJ1003 genome. **(A)** Statistical results of COG classification; the abscissa indicates the content of COG classification; the ordinate is the number of genes. **(B)** Clustering results of GO annotation; the abscissa indicates the content of each GO classification; the left side of the ordinate indicates the percentage of the number of genes, and the right side indicates the number of genes.

**FIGURE 3:**
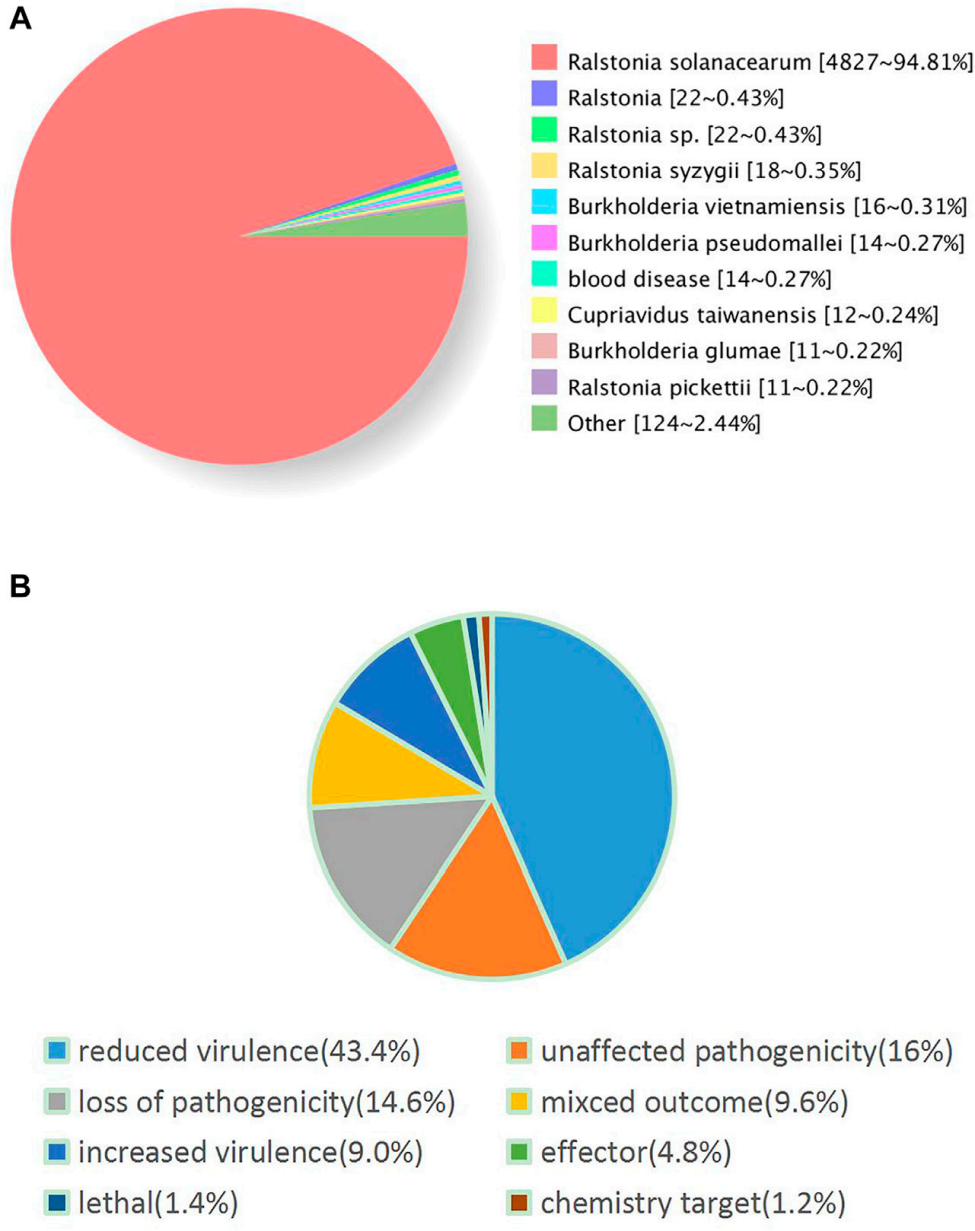
Enrichment analysis results based on the NR and PHI databases. **(A)** Statistical results of species distribution by the NR database. Different colors represent different homologous species. **(B)** The gene enrichment annotation to the PHI database. Different colors represent different types of disease-related genes.

Furthermore, we performed the functional annotation analysis of identified genes in the FJ1003 genome using the GO, NR, and COG databases. Among them, 3913 genes were annotated by the COG database, and 3855 genes by the GO database (Figure 2). In the NR database, 5064 genes were annotated, of which 4827 (94.81%) genes were highly conserved in *R. solanacearum* (Figure 3A). Similarly, the predicted results were used to BLAST against the PHI database to obtain the enrichment results (Figure 3B).

### Comparative analysis of the virulence factors in FJ1003

*R. solanacearum* has many virulence factors, among which the T3Es secreted by the T3SS, are considered the primary virulence factors (Landry et al., 2020). Based on the *R. solanacearum* T3Es (Peeters et al., 2013) and the NCBI database, we predicted 76 T3Es and compared them with T3Es from four other isolates (Table 2). Although RipBP and RS_T3E_Hyp6 were absent from the six other reference genomes, they were mainly found in the NCBI database searches of other *R. solanacearum* genomes. Notably, we found that five of the FJ1003 strain genes in the T3Es were absent from the model strain GMI1000. Although both CQPS-1 and FJ1003 infect tobacco, 19 T3Es were present in FJ1003 and were absent from CQPS-1 (Table 2).

**TABLE 2:** Summary of type III effector proteins genes (Coverage%/Identity%) in FJ1003 and other strains.

Effector name	FJ1003 gene ID	GMI1000 (I)	CQPS-1 (I)	UW700 (ⅡA)	UW551 (ⅡB)	CFBP3059 (Ш)	PSI07 (IV)
RipA1	1_1923	95/94	absent	absent	absent	absent	absent
	1_1924	100/98	absent	absent	absent	absent	absent
RipA2	2_580	100/99	100/100	101/78	101/78	100/92	100/79
RipA3	2_1308	100/98	100/99	100/70	100/68	72/94	100/81
RipA4	2_1309	100/99	100/99	100/76	93/52	92/92	100/80
RipA5	2_1465	100/99	99/99	100/79	100/78	98/95	100/82
RipAA	1_424	100/65	absent	100/76	100/75	100/75	100/80
RipAB	2_1338	100/99	100/100	absent	100/70	100/94	100/77
RipAC	2_1304	100/100	100/100	100/87	100/88	100/97	100/92
RipAD	2_449	95/99	absent	93/67	90/72	95/81	95/73
RipAE	1_106	100/98	100/100	absent	absent	102/52	100/76
RipAF1	2_1283	100/98	absent	absent	absent	absent	absent
RipAI	2_1299	100/99	100/99	99/81	99/80	100/95	99/85
RipAJ	1_1887	100/99	absent	100/70	100/68	100/75	100/69
RipAK	1_2138	100/100	100/99	absent	absent	absent	absent
RipAL	2_1184	absent	100/100	96/80	96/80	100/84	100/99
RipAN	2_1307	100/99	100/99	100/70	absent	100/89	92/74
RipAO	2_1341	100/98	100/100	absent	100/51	59/81	100/55
RipAP	2_122	79/98	100/100	100/79	100/78	100/84	absent
RipAQ	2_1348	100/99	100/100	absent	100/68	100/77	100/65
RipAR	2_143	100/99	100/99	97/62	100/64	100/87	96/74
RipAS	2_237	100/99	100/99	absent	97/51	absent	absent
RipAT	2_242	91/99	100/98	absent	100/61	absent	99/68
RipAU	2_313	100/98	100/98	99/75	absent	100/78	absent
RipAV	2_1192	100/99	100/98	absent	absent	100/88	absent
RipAW	2_332	100/96	79/98	100/63	83/54	83/83	85/67
RipAX1	1_3060	93/100	93/100	absent	94/73	94/78	absent
RipAY	2_1462	100/96	absent	100/58	absent	100/86	97/68
RipAZ1	2_436	100/99	72/99	absent	absent	72/93	72/91
RipBC	1_3046	100/100	100/100	100/94	100/93	100/99	100/98
RipB	1_33	100/99	absent	100/77	absent	100/81	100/80
RipBM	2_693	62/99	71/99	96/94	absent	71/93	71/94
RipBP	1_690	absent	absent	absent	absent	absent	absent
RipC1	2_146	100/99	absent	99/70	99/69	absent	100/96
RipC2	2_776	100/92	100/100	100/66	102/63	100/64	100/83
RipE1	1_3141	100/99	100/100	100/85	100/85	absent	100/80
RipF1	2_409	100/99	100/100	100/79	100/78	100/95	100/78
RipG1	2_1360	100/99	100/100	absent	absent	absent	100/50
RipG2	2_1134	100/98	100/100	absent	100/59	100/81	100/64
RipG3	2_557	100/95	100/100	100/56	98/56	100/85	97/69
RipG4	1_1357	100/99	83/52	100/60	100/56	93/51	100/51
RipG6	1_1148	100/97	100/80	absent	100/71	absent	98/73
RipG7	1_1149	100/75	100/100	98/67	absent	100/64	94/72
RipH1	1_1177	100/99	100/100	absent	absent	100/83	87/66
RipH2	2_692	100/97	100/98	100/64	absent	absent	99/55
RipH3	2_631	99/99	99/100	99/79	99/78	100/83	98/71
RipI	1_3248	100/100	100/99	100/82	75/85	100/87	100/93
RipJ	1_1919	100/93	100/100	absent	absent	absent	100/27
RipL	2_667	100/97	100/97	100/61	absent	absent	absent
RipM	1_1265	100/99	absent	99/71	100/72	100/88	100/80
RipN	2_44	100/99	100/99	100/73	100/77	100/90	89/74
RipO1	2_795	100/100	100/100	100/84	96/86	absent	absent
RipP1	1_181	100/100	100/100	100/95	100/95	100/100	100/95
RipP2	1_2971	100/99	absent	99/85	absent	absent	absent
RipP3	1_2935	61/100	absent	absent	absent	absent	absent
RipQ	2_184	100/100	70/97	absent	70/82	98/69	absent
RipR	2_189	100/99	absent	100/80	100/79	98/93	98/82
RipS1	1_3173	100/99	100/72	100/90	100/88	99/71	100/75
RipS2	2_223	98/99	absent	99/74	91/51	98/91	100/77
RipS3	2_1373	100/99	100/99	100/76	100/75	100/93	99/77
RipS4	1_1321	100/99	99/100	98/72	91/64	91/85	98/65
RipS5	2_772	100/99	100/100	100/79	100/70	100/86	100/76
RipS6	1_1917	100/96	96/51	96/50	absent	96/50	absent
RipS7	1_2037	100/99	100/99	absent	100/92	68/96	100/92
RipS8	1_1317	79/63	79/63	77/64	103/62	71/63	100/90
RipTAL	1_1343	100/99	100/100	absent	absent	absent	82/66
RipTPS	2_1191	100/99	100/99	absent	100/79	100/95	94/76
RipU	2_119	absent	absent	100/74	100/78	absent	100/77
RipV1	1_1141	100/100	100/100	100/64	100/65	100/85	100/71
RipW	1_2487	100/99	100/100	100/73	100/80	100/93	100/85
RipX	2_1339	100/98	100/100	absent	100/59	100/76	99/74
RipY	1_40	100/100	absent	100/78	100/74	absent	100/72
RipZ	2_1474	100/99	absent	97/68	98/68	absent	97/71
RS_T3E_Hyp12	2_670	100/99	100/100	100/82	100/83	90/96	100/90
RS_T3E_Hyp14	1_646	absent	84/100	absent	absent	absent	absent
RS_T3E_Hyp6	1_631	absent	absent	absent	absent	absent	absent
RS_T3E_Hyp8	2_900	100/100	100/100	100/94	100/95	100/99	100/97
	1_1388	100/100	100/100	96/92	96/92	100/98	96/91

The *R. solanacearum* T3SS family is considered to contain more than 20 structural genes. Therefore, we compared the FJ1003 T3SS with GM1000 T3SS and other virulence factors such as exopolysaccharides, cell wall degrading enzymes, and swimming ability-related genes (Supplementary Table S2). These virulence factors were conserved within the bacterial genome. The pehB, FliO, FliN, FliS, FliT, and FliK genes were 99% identical, while the remaining genes were 100% identical (Supplementary Table S2).

### Evolutionary relationship among species

**FIGURE 4:**
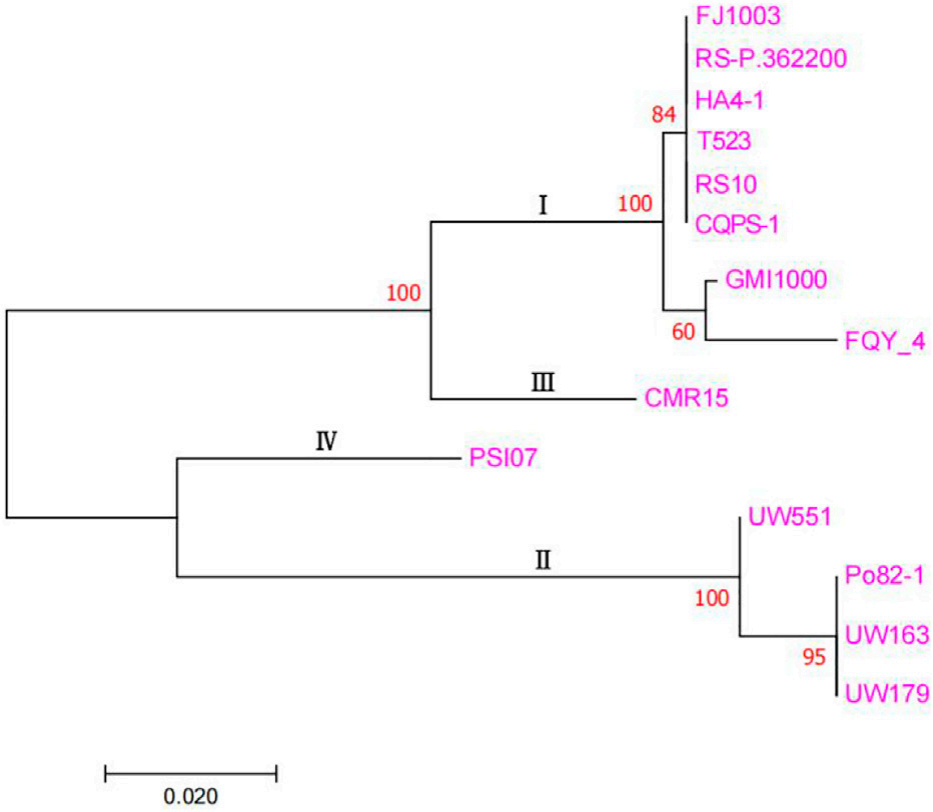
Molecular phylogenetic analysis between FJ1003 and other strains. This figure is mainly based on the sequence similarity of endoglucanase in different genomes. The percentage of tree in which the associated taxa were clustered together, is shown next to the branches. The tree is drawn to scale, with the corresponding branch lengths indicating the number of substitutions per site. The pink font on the far right is the name of the different species of *R. solanacearum*.

The detailed genome information of the genome sequences of the different phylotypes of *R. solanacearum* obtained from the NCBI database is presented in Supplementary Table S3. The phylogenetic tree (Figure 4) was drawn based on the sequence similarity among the endoglucanase genes from different genomes (Villa et al., 2005). Phylogenetic analysis revealed that the FJ1003 strain belonged to the same branch as the other phylotype I strain. Phylotypes I and III were closely related, while II and IV belonged to two independent branches, indicating that the “proteomics-genomics” method of classifying *R. solanacearum* into three classes is better suited for its classification. Despite belonging to the same phylotype, the genetic relationships between strains may vary depending on their host or geographical origin.

### Some genes are specific to the FJ1003 genome

**FIGURE 5:**
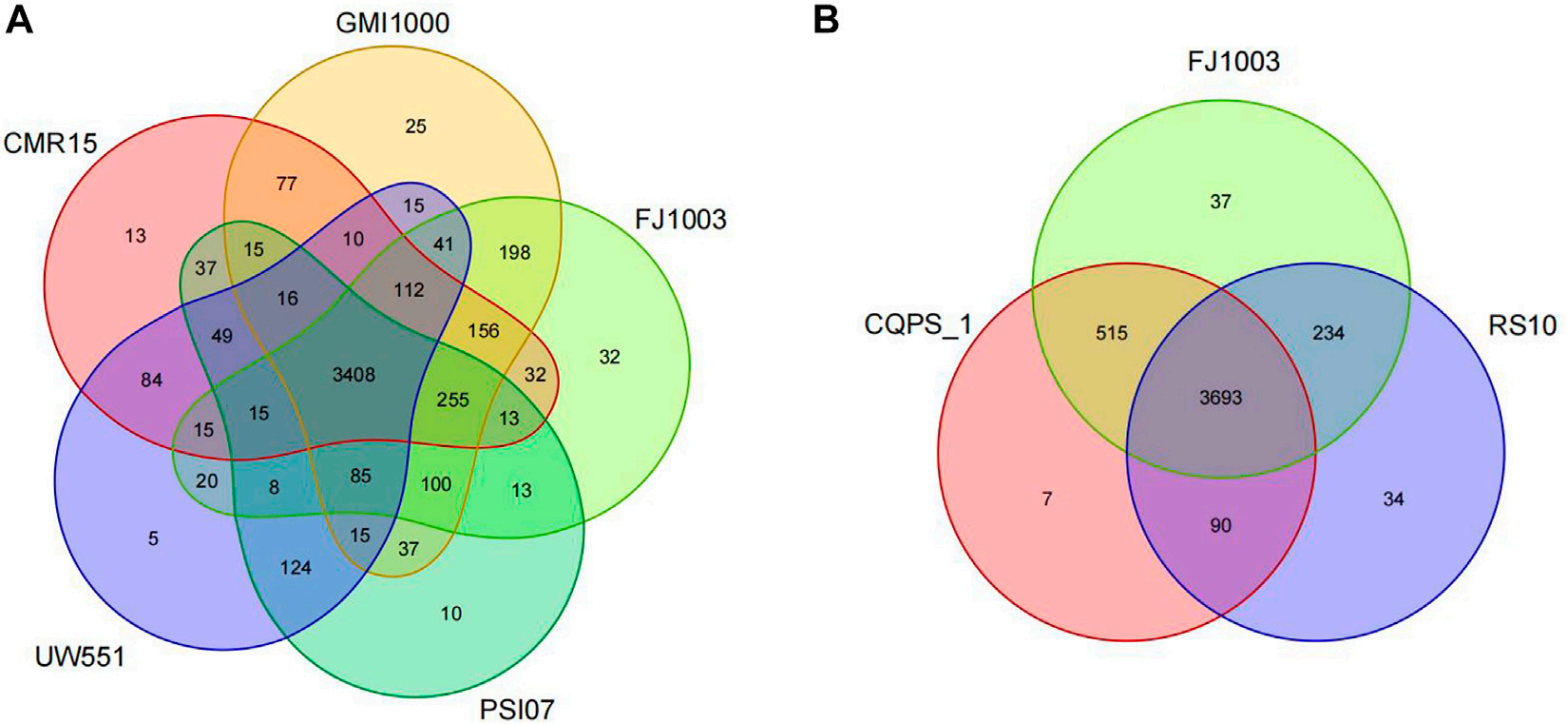
Venn diagrams showing the number of specific and shared genes among the *R. solanacearum* strain FJ1003 and other bacterial species. **(A)** Venn diagrams of the sequenced strains and reference strains from different phylotypes. **(B)** The hosts of the three strains are tobacco. Note: the overlapping regions in the figure indicate the number of gene families shared among the different species, while the regions that do not overlap with other species indicate the number of gene families specific to that species.

To find which genes are specific to the FJ1003 genome, we compared it with those of four other different strains from different phylotypes. The results showed that 73 genes were specific to the FJ1003 strain (Figure 5A; Supplementary Tables 4, S5-1). They included unknown functional proteins, transposable enzymes, twitching mobility, and plasmid replication initiation proteins. At the same time, we also analyzed the FJ1003 protein sequences with two other strains (CQPS-1 and RS10) with the same phylotype and host and found that 90 genes were unique to FJ1003 (Figure 5B; Supplementary Table S5-2). These unique genes included hypothetical proteins, hemagglutinin-like secreted proteins, membrane proteins, and integrases. Therefore, our results suggest that the genome of *R. solanacearum* is relatively complex and has high genetic diversity.

### Genomic collinearity analysis

**FIGURE 6:**
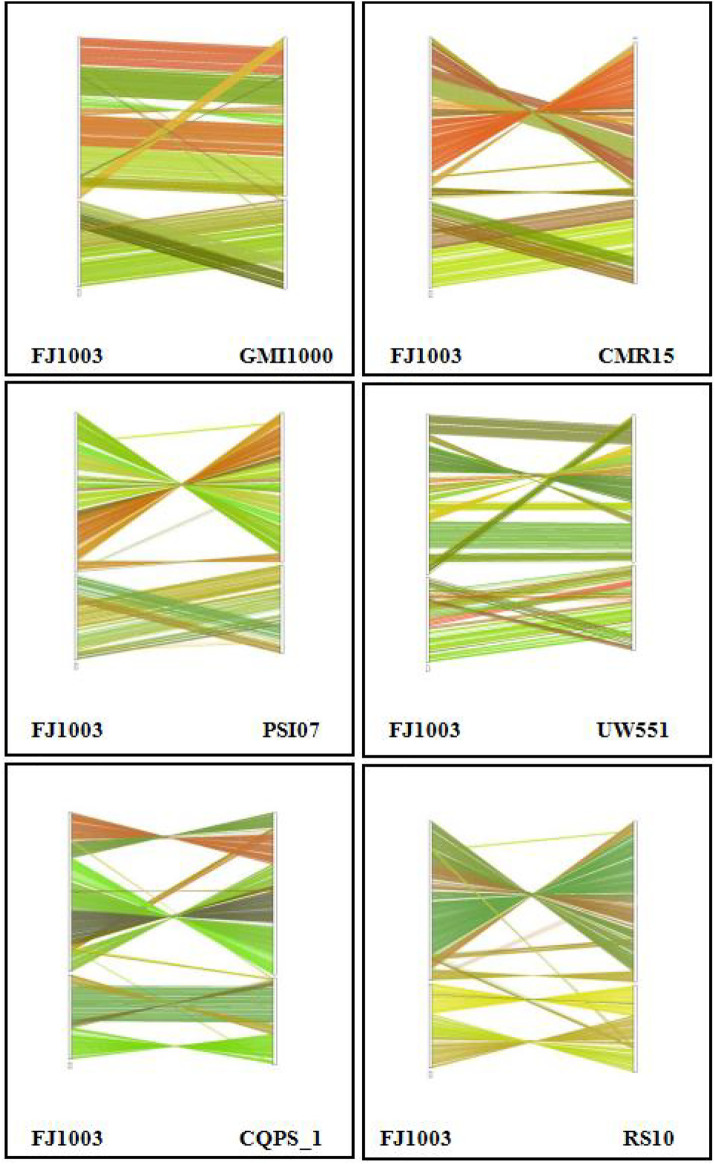
The collinear relationship between the FJ1003 strain and other strains. The genome location coordinates of the reference near-source species are measured on the left, while the assembled genome location coordinates are on the right. The lines in the figure represent the collinear regions between the two species. Lines of different colors represent different collinear areas between different chromosomes. The color of the lines is automatically assigned by the software.

The analysis of collinear genes with other reference genomes (GMI1000, CMR15, PSI07, UW551, and CQPS_1, RS10) showed that there was similarity among the whole genomes of the different strains of *R. solanacearum*. However, there were some cases of local non-collinearity, indicating that there were a few inverted and ectopic genome rearrangements (Figure 6). We also found some blank regions in the genome, indicating that there was dissimilarity among the sequences of the various strains, possibly with the specific genes of a particular strain.

### Horizontal gene transfer is widespread in the genome

In terms of microbial evolution, horizontal gene transfer (HGT) is referred to as the transfer of genetic material between non-parental organisms, either interspecifically or intraspecifically, making it a fairly widespread phenomenon with a vital role in the evolutionary processes (Villa et al., 2005; Shutian, 2020). A genetic island (GI) acts as an integrated moving element on the host population gennome by facilitating gene exchanges within or between species. Using HGT between different hosts, the acceptor strains obtain large DNA fragments, increasing their versatility and adaptability. During the process of bacterial HGT, mild bacteriophages transfer the donor genes to the recipient, and this transferred genetic fragment which is integrated into the recipient, is called a prophage. The FJ1003 genome contains 23 GIs and five Prophages (Supplementary Table S6). RipP2, RipP3, RipBP, and RS_T3E_Hyp14 are present in the prophages, whereas Rs_T3E_Hyp14 and RS_T3E_Hyp6 are located on the GI (Supplementary Table S6).

### RS-T3E-Hyp14 is involved in the pathogenicity of *Ralstonia solanacearum*

**FIGURE 7:**
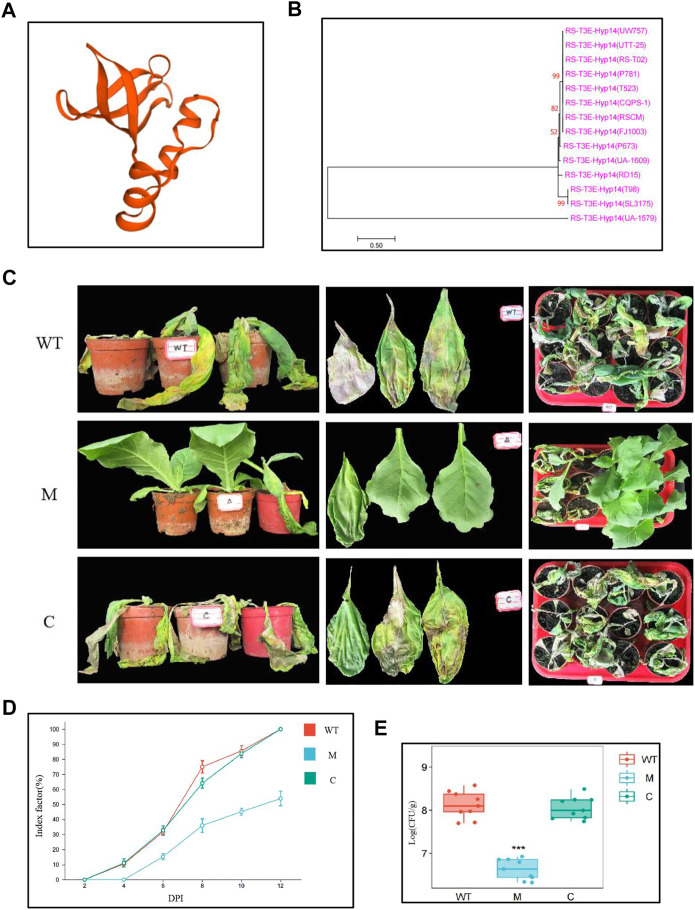
Functional identification of RS-T3E-Hyp14 **(A)** Tertiary structures of Rs_T3E_Hyp14 in FJ1003. **(B)** Molecular phylogenetic analysis of predicted RS-T3E-Hyp14 effectors. The phylogenetic tree was constructed by the maximum likelihood method. **(C)** Symptoms of tobacco after 8 days of inoculation with *Ralstonia solanacearum*. **(D)** Disease index of *Ralstonia solanacearum* at different time after inoculation **(E)** Colonization of *Ralstonia solanacearum* in tobacco stems after inoculation for 3 days. Values are means ± SE for three replicates. (WT: wild type strains FJ1003, M: mutant strains ΔRs_T3E_Hyp14, C: complementary strains CRs_T3E_Hyp 14ΔRs_T3E_Hyp14).

Notably, Rs_T3E_Hyp14 is present on prophage and GI both, proposing that this gene might have been acquired from other bacteria *via* HGT. We predict the tertiary structures of Rs_T3E_Hyp14 using SWISS-MODEL (https://swissmodel.expasy.org/interactive/PW9vQ4/models/) (Figure 7A) and found it to be a typical feature of T3E AvrRpt2 (a C70 family cysteine protease) from *Erwinia amylovora*. At the same time, through NCBI and T3E database comparison, we found that 14 strains of *Ralstonia solanacearum* had Rs_T3E_Hyp14 gene, and by blastp analysis, the Rs_T3E_Hyp14 gene of five strains was 100% similar to that of FJ1003 (Supplementary Table S7). We constructed an evolutionary tree of Rs_T3E_Hyp14 of these 14 strains using the ML method and found that the genetic distances of Rs_T3E_Hyp14 in most of the phylotype I strains were similar (Supplementary Table S7, Figure 7B). There is no report on the function of RS-T3E-Hyp14. The pathogenicity of *R. solanacearum* on tobacco is significantly reduced after we knockout RS-T3E-Hyp14, and the colonization of *R. solanacearum* on a host is also affected (Figures 7C–E). The phenotype of complementary strains is consistent with that of wild strains (Figures 7C–E). These results suggest that RS-T3E-Hyp14 is a virulence factor in the pathogenic process of *R. Solanacearum* FJ1003, which affects its pathogenicity.

## Discussion

Bacterial wilt caused by *R. solanacearum* is a common bacterial disease affecting tobacco production. It is rapidly spreading and very destructive during all growing stages of tobacco (Lin, 2018). *R. solanacearum* demonstrates high genetic diversity and host specificity. FJ1003 is a strain isolated from tobacco and has not been reported to affect other host species. To date, 291 strains of *R. solanacearum* have been sequenced and assembled, among which both the RS10 and CQPS-1 strains were isolated from tobacco. Upon comparing the protein sequences of FJ1003 and four other reference strains, 73 genes were found to be FJ1003-specific, while the other two tobacco isolates (RS10 and CQPS-1) all had their specific genes. These results may further prove that *R. solanacearum* is genetically diverse. Moreover, subsequent whole-genome sequencing of the *R. solanacearum* isolated from tobacco from different geographical regions could shed light on this point of view for future investigations.

Using the syringe-like T3SS, *R. solanacearum* can inject multiple toxic effector proteins into the host cells, thereby disturbing the host immune system and finally causing host disease. Effector proteins have a wide variety of functions, including the effector proteins (RipM, RipS1, RipQ, RipG3, RipD, RipAD, and RipAU) that inhibit the plant immune response triggered by the bacterial flg22 (Landry et al., 2020). Upon injection of the bacterial effector protein RipTPS into the plant cell, trehalose-6-phosphate is synthesized in planta to provide nutrition for the reproduction of *R. solanacearum* in the host (Poueymiro et al., 2014). RipN can adversely alter the NADH/NAD + ratio and inhibit the immune response triggered by pathogen-associated molecular patterns (PAMPs) (Sun et al., 2019). RipI can modulate plant metabolism to enhance γ-aminobutyric acid (GABA) production (a signalling molecule) in plant cells, which *R. solanacearum* uses as a nutrition source during its reproductive process in plants, thus finally causing plant disease (Xian et al., 2020). Few effector proteins, like RipP1 and RipP2, function as avirulence proteins (Landry et al., 2020). Thus, these results indicate that FJ1003 contains 76 T3Es, with the function and pathogenesis of most effector proteins still being unknown, and further research is needed. The functional annotation by the PHI database indicates that some effector proteins related to the host-pathogen interaction may play a key role in the pathogenesis of *R. solanacearum*.

HGT, the exchange of genetic material between individuals, is an important factor behind microbial evolution and diversity (Syvanen, 1985). We detected five prophages (a total of 316 genes) and twenty-three GIs (a total of 252 genes) in the *R. Solanacearum* FJ1003 genome. Interestingly, RS-T3E-Hyp14 is present in both prophages and genomic islands, suggesting that this gene is associated with HGT. The function of RS-T3E-Hyp14 has not been reported. We identified the function of RS-T3E-Hyp14 and found that it could significantly reduce the pathogenicity of *Ralstonia solanacearum* and play an important role in the pathogenic process of *R. solanacearum*. Further studies on the pathogenesis of RS-T3E-Hyp14 and its interaction with tobacco are needed.

Most prokaryote genomes contain the CRISPR-Cas system, mainly containing CRISPR sequences (Louwen et al., 2014). This system is developed throughout the long-term struggle of prokaryotes against harmful foreign substances. It is a mechanism for protecting prokaryotes from harmful foreign agents. The current study predicted three copies of 100-bp CRISPR sequences in the FJ1003 genome. The widespread presence of HGT and CRISPR sequences in *R. solanacearum* may be one of the reasons for its wide host range, biological diversity, and strong pathogenicity.

The PHI database contains the pathogenic genes, virulence genes and effector protein genes of pathogens that have been verified by experiments or reported in the literature (http://www.phi-base.org/index.jsp). Inactivation or reduced expression of most of these genes can possibly result in the reduction or even the complete loss of the pathogenic ability of the pathogen in the corresponding host. The annotation of 869 genes in the FJ1003 genome by the PHI database falls under seven different modules (reduced virulence, unaffected pathogenicity, loss of pathogenicity, mixed outcome, lethal, increased virulence, effector, and chemistry target). These genes may play different roles in the host-pathogen interaction (ZANG et al., 2021). Therefore, further research is needed to provide an important theoretical basis for the screening and functional verification of pathogenic proteins.

## Conclusion

In this study, we sequenced the genome of a plant pathogen, *Ralstonia Solanacearum* strain FJ1003, isolated from Fujian province, China. Analysis showed that the genome consists of a chromosome, a megaplasmid, and a small plasmid. The evolutionary analysis provided that FJ1003 belonged to phylotype I and this strain contained 76 T3Es. Horizontal gene transfer was widespread in this strain, and the RS-T3E-Hyp14 existed in both prophages and genomic islands. This study is the first report on the genetic functions of RS-T3E-Hyp14. The knockout mutants of RS-T3E-Hyp14 significantly reduced the pathogenicity of *R. solanacearum* and weakened its colonization in the host. Results provide valuable information for the control of bacterial wilt in tobacco plants.

## Data Availability

The genome sequences have been deposited in the NCBI SAR database under the accession number PRJNA780084, available at https://www.ncbi.nlm.nih.gov/bioproject/?term=PRJNA780084.
