# Supplementary material for: Comprehensive genome sequence analysis of the devastating tobacco bacterial phytopathogen Ralstonia solanacearum strain FJ1003
Source: Front Genet. 2022 Aug 22;13:966092. doi: 10.3389/fgene.2022.966092 (PMC9441608; doi:10.3389/fgene.2022.966092)
Supplement: Supplementary file 1 [file DataSheet1.ZIP › Supplementary/Supplementary Figure 1.pdf]

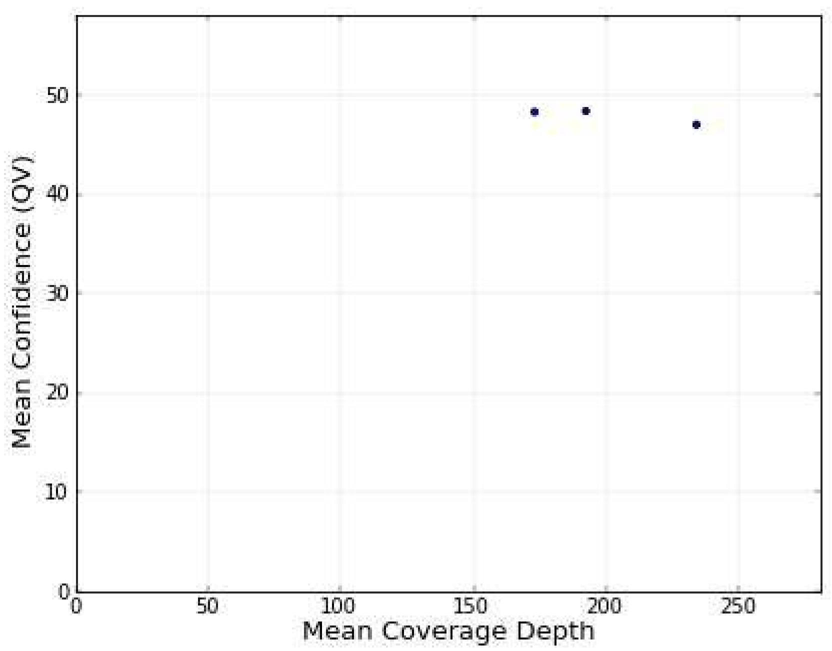

Supplementary Figure 1. Statistical map of genome alignment efficiency. Abscissa represents the average coverage of sequencing. The ordinate represents the sequencing accuracy, and the calculation formula is as follows: QV=-10*log10 (p), where p is the sequencing base error rate. For example, the accuracy of QV20 is 99% and the accuracy of QV50 is 99.999%.
