# Supplementary material for: Comprehensive genome sequence analysis of the devastating tobacco bacterial phytopathogen Ralstonia solanacearum strain FJ1003
Source: Front Genet. 2022 Aug 22;13:966092. doi: 10.3389/fgene.2022.966092 (PMC9441608; doi:10.3389/fgene.2022.966092)
Supplement: Supplementary file 1 [file DataSheet1.ZIP › Supplementary/Supplementary Figure 2..docx]

| 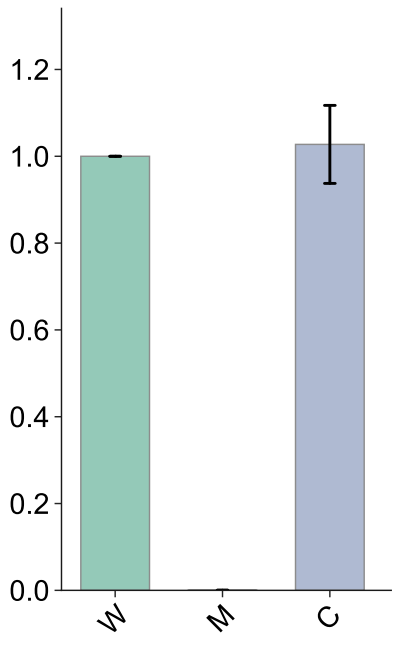  **Relative expression level** |
| --- |
| **Supplementary Figure 2. Expression of RS-T3E-Hyp14 in different types of *Ralstonia solanacearum*. Values are means ± SE for three replicates.** (W: wild type strains FJ1003, M: mutant strains ΔRs_T3E_Hyp14, C: complementary strains CRs_T3E_Hyp 14ΔRs_T3E_Hyp14). |
